# Macular scar secondary to congenital toxoplasmosis

**DOI:** 10.11604/pamj.2016.24.325.8981

**Published:** 2016-08-24

**Authors:** Sophia El Hamichi, Abdelbarre Oubaaz

**Affiliations:** 1 Military Hospital Mohammed V of Rabat, Rabat, Morocco

**Keywords:** Macular scar, congenital toxoplasmosis, strabismus

## Image in medicine

An 8 years old girl suffers from strabismus since her first months of life. Her visual acuity was very low and could only see fingers moving in her left eye. Her left eye fundus showed a chorioretinal scar in the macula due to congenital toxoplasmosis. The biological findings proved the diagnosis of congenital toxoplasmosis. Functional prognosis of macular scars is very dark.

**Figure 1:**
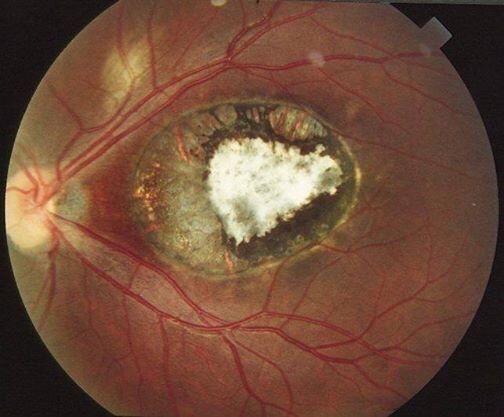
Retinography showing a macular retinochoroidal scar secondary to toxoplasmosis

